# Modulation of entorhinal cortex–hippocampus connectivity and recognition memory following electroacupuncture on 3×Tg-AD model: Evidence from multimodal MRI and electrophysiological recordings

**DOI:** 10.3389/fnins.2022.968767

**Published:** 2022-07-29

**Authors:** Bingbing Lin, Lanlan Zhang, Xiaolong Yin, Xiaocheng Chen, Chendong Ruan, Tiecheng Wu, Zhizhen Liu, Jia Huang

**Affiliations:** ^1^College of Rehabilitation Medicine, Fujian University of Traditional Chinese Medicine, Fuzhou, China; ^2^TCM Rehabilitation Research Center of State Administration of Traditional Chinese Medicine (SATCM), Fujian University of Traditional Chinese Medicine, Fuzhou, China; ^3^National-Local Joint Engineering Research Center of Rehabilitation Medicine Technology, Fujian University of Traditional Chinese Medicine, Fuzhou, China; ^4^Key Laboratory of Orthopedics & Traumatology of Traditional Chinese Medicine and Rehabilitation, Ministry of Education, Fuzhou, China

**Keywords:** Alzheimer’s disease, electroacupuncture, recognition memory, functional connectivity, electrophysiology

## Abstract

Memory loss and aberrant neuronal network activity are part of the earliest hallmarks of Alzheimer’s disease (AD). Electroacupuncture (EA) has been recognized as a cognitive stimulation for its effects on memory disorder, but whether different brain regions or neural circuits contribute to memory recovery in AD remains unknown. Here, we found that memory deficit was ameliorated in 3×Tg-AD mice with EA-treatment, as shown by the increased number of exploring and time spent in the novel object. In addition, reduced locomotor activity was observed in 3×Tg-AD mice, but no significant alteration was seen in the EA-treated mice. Based on the functional magnetic resonance imaging, the regional spontaneous activity alterations of 3×Tg-AD were mainly concentrated in the accumbens nucleus, auditory cortex, caudate putamen, entorhinal cortex (EC), hippocampus, insular cortex, subiculum, temporal cortex, visual cortex, and so on. While EA-treatment prevented the chaos of brain activity in parts of the above regions, such as the auditory cortex, EC, hippocampus, subiculum, and temporal cortex. And then we used the whole-cell voltage-clamp recording to reveal the neurotransmission in the hippocampus, and found that EA-treatment reversed the synaptic spontaneous release. Since the hippocampus receives most of the projections of the EC, the hippocampus-EC circuit is one of the neural circuits related to memory impairment. We further applied diffusion tensor imaging (DTI) tracking and functional connectivity, and found that hypo-connected between the hippocampus and EC with EA-treatment. These data indicate that the hippocampus–EC connectivity is responsible for the recognition memory deficit in the AD mice with EA-treatment, and provide novel insight into potential therapies for memory loss in AD.

## Introduction

Alzheimer’s disease (AD), the most common cause of dementia, is a progressive neurologic disorder that irreversible anatomical, cognitive, and behavioral deficits leading to affect a person’s ability to function independently. As the disease processes, patients present with varying manifestations of cognitive decline including memory loss, thinking, reasoning skills, and language impairment (Wattmo and Wallin, 2017; Kamiya et al., 2018; Lane et al., 2018). The memory problem is a core symptom, notably, recognition memory is one of the first warning signatures of AD (Russo et al., 2017; Goldstein et al., 2019). Although there is no currently approved cure for AD, promising treatments emerge including trials aimed at preventing memory loss in early AD.

Increasing clinical and experimental studies have confirmed the efficacy of electroacupuncture (EA) on cognitive dysfunction (Wang et al., 2020; Yu et al., 2020; Yin et al., 2022). EA has been recognized as a crucial treatment in traditional Chinese medicine (TCM), which applies the inserted needles with an electric stimulating current to the acupoints. Clinical trials have shown that EA-treatment improved cognitive outcomes in early and mid-stage AD patients, and notably on memory function in AD prodromal patients (Deng and Wang, 2016; Jia et al., 2017; Lin et al., 2022). A review summarized that EA at acupoints such as Baihui (DU20), Shenting (DU24), and Neiguan (PC6) effectively improve the score of cognitive tests in patients with cognitive impairment, respectively (Yin et al., 2021). Our previous clinical study showed that EA at DU20/DU24 could effectively improve the score of MMSE and MoCA in patients with cognitive impairment (Jiang et al., 2016). Furthermore, animal studies also demonstrated altered behaviors in memory-loss rodents after EA-treatment (Huang et al., 2016). It has been described possible potential mechanisms such as regulation of synaptic plasticity and neuroinflammation in rodent models of AD. Besides, synaptic dysfunction is thought to lead to a chaos of functional brain activity in early AD (Sperling et al., 2011). Previous clinical and animal studies reported that EA-induced cognitive enhancement is closely related to the hippocampus (Hip) (Wang et al., 2014; Zheng et al., 2018, 2021; Li et al., 2021). Moreover, it could be speculated that in addition to hippocampal synaptic plasticity, EA-treatment could have other neuromodulation mechanisms that regulate memory function.

Compelling evidence demonstrated that altered recognition memory in both cognitive impairment patients and animals is potentially associated with changes in regional spontaneous activities and neural connectivity, including the hippocampal-cortex network (Matura et al., 2014; De Marco et al., 2017; Tanimizu et al., 2018; Salimi et al., 2022). Previous studies found that EA not only increased the neuronal activity of the hippocampus in mild cognitive impairment (MCI) patients but also enhanced the functional connections between brain regions (Tan et al., 2017). In addition to the functional connectivity (FC) and neuronal activity, the neuronal fiber connections revealed by diffusion tensor imaging (DTI) can also reflect the structural connectivity between brain regions. Studies showed that AD patients have lower nerve fiber density in the hippocampus than healthy elderly people, and it is positively correlated with memory behavior (Mayo et al., 2017, 2018). In the AD early stage, a gradual disruption of synapse and neuronal connections appear in parts of the brain regions dedicated to object recognition memory, particularly the entorhinal cortex (EC) and Hip (Braak et al., 2011). Evidence showed that functional and structural alterations in EC and Hip can be suggested as potential biomarkers for early AD (Devanand et al., 2007). Generally accepted that the EC–Hip interaction is pivotal for memory processing. However, the role of neuronal activities and neural connectivity alterations in the EC–Hip network in EA-induced memory recovery of AD remains unknown.

Therefore, the objective of the present study was to elucidate a comprehensive mechanism for the effects of EA on recognition memory deficits in AD. We hypothesized that EA treatment can regulate neuronal activities and neural connectivity in the EC–Hip network, and improve recognition memory. In this study, the open field test (OFT) and novel object recognition test (NORT) were performed after treatment, according to the time point of memory enhancement by EA-treatment in previous animal studies. We then employed 7.0 T resting-state functional magnetic resonance imaging (rs-fMRI) to observe where and to what extent neuronal activities take place in the brain after treatment. The hippocampus, which is thought to be the hub of memory-related networks in AD, showed changes with EA-treatment. To investigate if synaptic neurotransmission of the hippocampus was influenced by the EA-treatment, the spontaneous postsynaptic currents (sEPSC) recording at the hippocampal CA1 was measured. Finally, FC and DTI tracking were applied to detect the connections on hippocampal-related networks. The knowledge on how EA modulates neuronal activities and neural connectivity in AD mice will provide insight toward developing an effective strategy to overcome memory impairments.

## Material and methods

### Animals

Five months 3×Tg-AD mice and age-matched littermate wildtype-controls were used in the experiments (AD: *n* = 30, wildtype: *n* = 10). Male 3×Tg-AD transgenic mice express mutant human APPSwe, human PS1 (M146V) and mutant tau (P301L), thus mimicking pathological features of human AD. The offspring were genotyped by polymerase chain reaction (PCR) analysis using tail DNA to confirm the presence of mutant genes. The mice were housed in cages of up to 5 and kept in the standard environmental conditions, under a 12-h light/dark cycle and room temperature, with food and water provided *ad libitum*. All procedures using mice were approved by Fujian University of Traditional Chinese Medicine Animal Experiment Ethics Committee and Authority (ethical animal permission no. FJTCM IACUC2019031).

### Electroacupuncture stimulation

**FIGURE 1:**
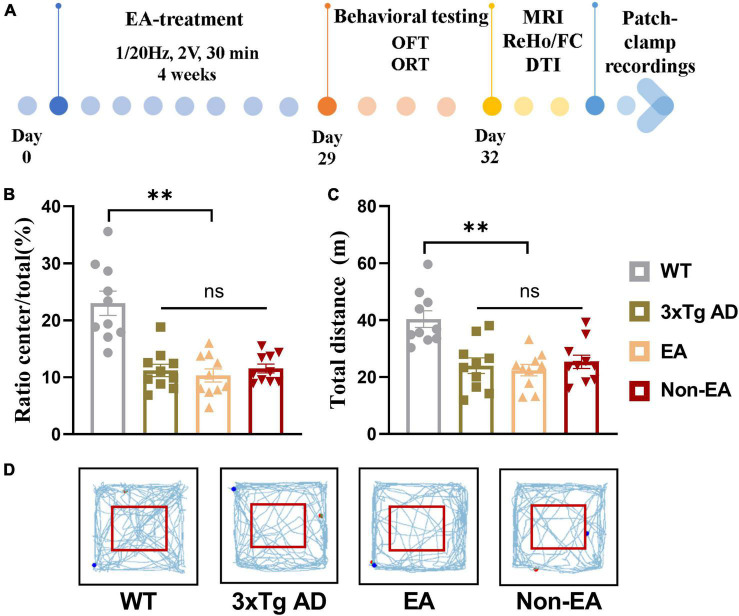
Effect of EA-treatment on locomotor activity in 3×Tg AD mice. **(A)** A schematic of the experimental design. Quantification of behavioral results in open field test (*n* = 10 in each group), involving **(B)** the percentage of time in the central zone, **(C)** the total distance traveled throughout the field, **(D)** and the trajectories of mice in the open field. All data represent the mean ± SEM. **P < 0.01; ns, there was no significant difference.

The homozygous 3×Tg-AD mice were randomly assigned to three groups by the random digital table: EA-treated (EA group), non-acupoint EA-treated (Non-EA group), and control untreated mice (3×Tg AD group), age-matched littermate wildtype-controls was assigned to the WT group. The EA stimulation was performed with a disperse-dense-mode stimulation at an intensity level of 1 mA and a frequency of 1/20 Hz by using a stimulator (model G6805, Suzhou Medical Appliance Factory, Shanghai, China) for 30 min daily, 5 consecutive days weekly, 4 weeks. Before stimulation was initiated, the torso of the mice was fixed to restrict excessive mobility. EA stimulation was applied at either the DU20(Baihui)/DU24(Shenting) or the non-acupoints by inserting the 0.25 × 13 mm stainless steel acupuncture needle (Hwato, China) about 2–3 mm depth in each site. For the EA group, the mice received EA stimulation at DU20 and DU24 acupoints. The DU20 acupoint is located at the midline of the head, approximately midway on the line connecting the apices of the auricles. The DU24 acupoint is located at 2 mm directly above the midpoint of the mouse’s eyes in Supplementary Figure 1A. For the Non-EA group, the EA stimulation was applied at non-acupoints for acupoint control, which were chosen to avoid the DU meridian and nearby acupoints. The bilateral non-acupoints are located at the bilateral hypochondrium, 2 cm above the posterior superior iliac spine and 3 cm lateral to the spine in Supplementary Figure 1B. The mice in the 3×Tg AD group and WT group (littermate controls) were fixed in the same way for 30 min without EA stimulation. The study timeline with all manipulations is presented in Figure 1A.

### Behavioral and cognitive testing

The locomotor activity was determined by OFT. Recognition memory was assessed in a NORT that involves exploration activity that responds to novelty. The locomotor activity was assessed 1-day post-treatment, and NORT conduct 2-days post-treatment.

All the tests were recorded using a digital USB camera (Super HAD CCD; SONY, Japan). The video tracking was processed using a video tracking system and SuperMaze analysis system (XR-Xmaze, Shanghai Soft maze Information Technology Co., Ltd., China). The testing apparatuses of OFT and NORT were thoroughly cleaned with 10% ethanol between animals. The measurements were carried out in a quiet environment with the background noise controlled at ≤60 dB.

### Locomotor activity

After habituation, the animal was placed into one corner of an open field arena (50 × 50 × 40 cm) that was divided into peripheral and central zones, and locomotor activity was measured for 5 min by total distance traveled and time spent in the central zones.

#### Novel object recognition testing

Novel object recognition test was conducted in the arena where the OFT was performed the day before, and the habituation phase was considered negligible. During the training phase, the animal was familiarized with two identical objects for 10 min, and then was returned to its home cage for 24 h, after which the testing phase took place. During the recognition phase, the animal was placed back into the arena which contain one of familiar objects and a novel object for 10 min. The choice to explore the novel object was considered to be the use of learning and long-term recognition memory. Exploration times and times are counted for the nose/head or paws entering the object area (about 2 cm around the object). The recognition index (time) was calculated as time spent with the novel object/time spent with both objects. The recognition index (number) was calculated as number of exploring the novel object/numbers of exploring both objects ×100.

### Magnetic resonance brain imaging protocols

Magnetic resonance imaging was acquired by Bruker 7.0T scanner (MiniMR-60 MRI system 7.0 T), and rs-fMRI and DTI were performed 10-day post-treatment. During the scan, an animal physiological detector (SurgiV et V3395TPR, Smiths Medical, United States) was used to monitor the body temperature, blood oxygen saturation and respiration of mice. The temperature control and ventilation system were used to maintain the stability of the physiological state throughout the experiment. Standardized anesthesia protocols and animal monitoring procedures were administered. Mice were initially anesthetized with 4% isoflurane (RWD, China) in a 1:4 O_2_ air mixture for 4 min, and then were placed on an MRI-compatible stand equipped with a hot water flow bed to maintain the animal temperature at 36.5 ± 0.5°C throughout the measurements. Mice were immobilized with ear rods and mechanically ventilated by a ventilator at a rate of 80 beats/min at a flow rate of 1.8 ml/min and a flow rate of 0.5-0.75% isoflurane. The blood oxygen saturation was kept above 95% throughout the scan, and the respiratory rate was 80–120 beats/min. Data analysis was performed by an experimenter blind to group information.

#### Functional magnetic resonance imaging acquisition

The acquisition parameters were as follows: T2-weighted images (T2WI) using TurboRARE sequence: TR/TE = 4,200/35 ms, field of view = 20 × 20 mm, averages = 4, image size = 256 × 256, slices = 30, slice thickness = 0.5 mm; rs-fMRI using an echo-planar imaging (EPI) sequence: TR/TE = 2,000/10.28 ms, field of view = 20 × 20 mm, repetitions = 200, image size = 64 × 64, slices = 30, slice thickness = 0.5 mm. The original image data was exported to DICOM format data by Paravision 6.0.1 software.

The rs-fMRI image data was carried out using Statistical Parametric Mapping (SPM8^1^) and the Data Processing Assistant for Resting-State fMRI (DPARSF^2^) software package (Wu et al., 2017). For the ReHo data, pre-processed with the following major steps: (1) The data of the first 10 time points were discarded for time calibration. (2) Time-slice correction. (3) Head movement correction. (4) Spatial standardization. (5) Detrend and filter: images were removed linear trend and the 0.01–0.08 Hz frequency band is used to perform time-space mean filtering on the data. The FC data preprocessing process is the same as that of ReHo, but spatial smoothing is performed in advance. Where the head movement translation is greater than 1.5 mm or the rotation angle exceeds 1.5°, the data is rejected.

#### ReHo analysis

ReHo is a local measure of the temporal similarity between a particular voxel and its neighbors, and is used to assess the strength of functional synchronization in local brain regions (Zang et al., 2004). Use Kendall’s coefficient of concordance (KCC) to define the ReHo value of the center voxel. Individual ReHo maps were generated by calculating the KCC value of the time series for a given voxel with its nearest neighbors, and smoothened by Gaussian kernel with a 1.5× voxel size Full Width of Half Maximum (FWHM) to improve the signal-to-noise ratio (SNR) for further statistical analysis. Voxel-wise statistics were performed with one-way analysis of variance (ANOVA) to determine the difference among all groups. The threshold for statistical significance was set at *P* = 0.005.

#### Seed-to-seed functional connectivity

According to the analysis results of ReHo, the bilateral EC and hippocampus were selected as regions of interest (ROIs). The average time series of the time series of all voxels in the region of interest was extracted, and then the correlation coefficient of the time series of signals between the EC and the hippocampus was calculated to obtain the FC strength, which was subjected to one-way ANOVA.

#### Diffusion tensor image acquisition

Diffusion tensor image scanning equipment and environment are the same as those of resting-state imaging. Unlike the resting-state imaging scans, the ventilator was mechanically ventilated at a rate of 80 beats/min with a flow rate of 3 ml/min and a flow rate of 1–1.5% isoflurane. The blood oxygen saturation was kept above 95% throughout the scan, and the respiratory rate was controlled at 60–90 beats/min to reduce head movement.

The acquisition parameters were as follows: a standard gradient-echo echo planar imaging sequence was used: TR = 12,000 ms, TE = 33 ms, averages = 2, FOV = 20 mm × 20 mm, image size = 96 × 96, slices = 30, slice thickness = 0.5 mm, *b* values = 1,000 s/mm^2^, number of diffusion directions = 30.

DICOM format data was converted to NIfTI format data by MRIcron software. Fiber tracking was performed using the TrackVis image processing software and the FACT algorithm. By registering the mouse standard brain template to the b0 image, the conversion relationship from standard space to individual space is obtained. Using this transformation relationship, the mask images of the bilateral EC and bilateral hippocampus in the Paxinos atlas space were transformed into the individual space, that is, the corresponding EC and hippocampus mask images of each mouse individual space were obtained. They were then identified as ROIs, and the number of nerve fibers between the two ROIs in each group of mice was measured. The statistical analysis of nerve fibers among groups was subjected to one-way ANOVA.

### Patch-clamp recordings

Mice were deeply anesthetized with isoflurane (RWD, China) and the brains were rapidly removed. The brain was placed in ice-cold artificial cerebrospinal fluid (ACSF) saturated with an oxygen gas mixture (95% O_2_ + 5% CO_2_) for 30 s. ACSF main components (in mM): 124 NaCl, 3.3 KCl, 1.2 KH_2_PO_4_, 26 NaHCO_3_, 2.5 CaCl_2_, 1.2 MgSO_4_, 10 glucose (pH = 7.3). Coronal slices of 400 μm were cut with a vibrating slicer (VT 1000s; Leica) and kept at 20–25°C in ACSF saturated with 95% O_2_/5% CO_2_ for at least 1 h before recording.

The deep layer neurons in hippocampal CA1 neurons were viewed under a Nikon microscope equipped with a 40× water immersion objective and a high-performance charge-coupled device camera. The pipette (6–9 MΩ) was filled with an internal solution (in mM, 124 NaCl, 2.5 KCl, 1.2 NaH_2_PO_4_, 24 Na HCO_3_, 5 HEPES, 12.5 D-glucose, 2.0 CaCl_2_, 1.5 MgSO_4_, QX-314 1.5 mmol/L). GABA receptor blocker (picrotoxin, 50 μM, Sigma) was added to the perfused ACSF. The sEPSC recordings were made at a holding membrane potential of −70 mV. Voltage and currents were recorded with a 700A Axon patch amplifier (Axon Instruments, United States). Data were digitized (Digidata 1322 digitizer; Molecular Devices, United States) and stored (Clampfit, version 10.6, Axon, United States). Off-line data of sEPSC analysis was performed using the Mini Analysis software (6.0, Synaptosoft, United States) and Origin 8.0 (OriginLab Corporation, United States). The amplitude and frequency of sEPSC were analyzed.

### Statistical analysis

The data are presented as the mean ± standard error of mean (SEM). To compare discrepancies among groups, data from behavioral tests and sEPSC were performed statistical analysis by one-way ANOVA using SPSS 26.0, followed by the *post hoc* test. If the variances were homogeneous, the Turkey-Kramer HSD test was used as a *post hoc* test. If the variances were not homogeneous, the Dunnett’s T3 test was used as a *post hoc* test. The data from ReHo, FC and DTI were tested for correlations with ORT data using Pearson linear regression analysis, respectively. *P* < 0.05 was considered statistically significant.

## Results

### Effects of the electroacupuncture treatment on behavioral and cognitive impairments

As shown in Figure 1, the results obtained in the OFT 1-day post-treatment revealed a significant difference in the wildtype-control mice and untreated 3×Tg-AD mice. There was no significant difference between the untreated and treated 3×Tg-AD mice in exploratory activities, even EA or Non-EA treatment.

**FIGURE 2:**
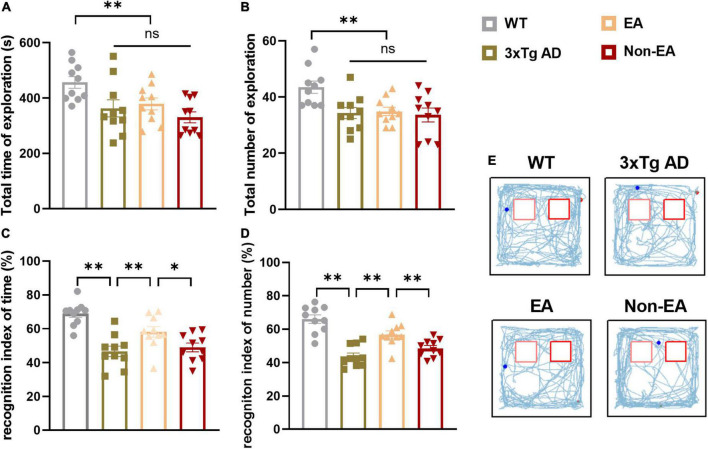
Effect of EA-treatment on recognition memory in 3×Tg-AD mice. Quantification of exploratory activity in ORT (*n* = 10 in each group), involving **(A)** the total time spent exploring, and **(B)** the total number of visits to both objects. Quantification of recognition memory, involving **(C)** the recognition index of time, **(D)** and the recognition index of number during probe test. **(E)** The trajectories of mice during probe test in the ORT. All data represent the mean ± SEM. *P < 0.05; **P < 0.01; ns, there was no significant difference.

Analysis of recognition memory in the NORT performed 2–3-days post-treatment is summarized in Figure 2. The exploratory activity of the 3×Tg-AD mice was reduced, including the total time spent exploring and the total number of visits to both objects (Figures 2A,B). However, EA or Non-EA treatment did not affect it, supporting the results obtained in the OFT. As observed in a representative tracking figure during the test probe (Figure 2E), the wildtype-control mice remained for a longer period close to the novel object as compared with that of the untreated 3×Tg-AD mice. The time spent exploring the novel object and the number of visits presented a tendency to ameliorate with the EA-treatment. Furthermore, differences between the untreated and EA-treated 3×Tg-AD mice were significant but Non-EA-treatment presented no difference in the recognition indexes, which were calculated from the total time spent exploring two objects and the number of visits (Figures 2C,D).

### Effects of the electroacupuncture treatment on regional spontaneous activity

**FIGURE 3:**
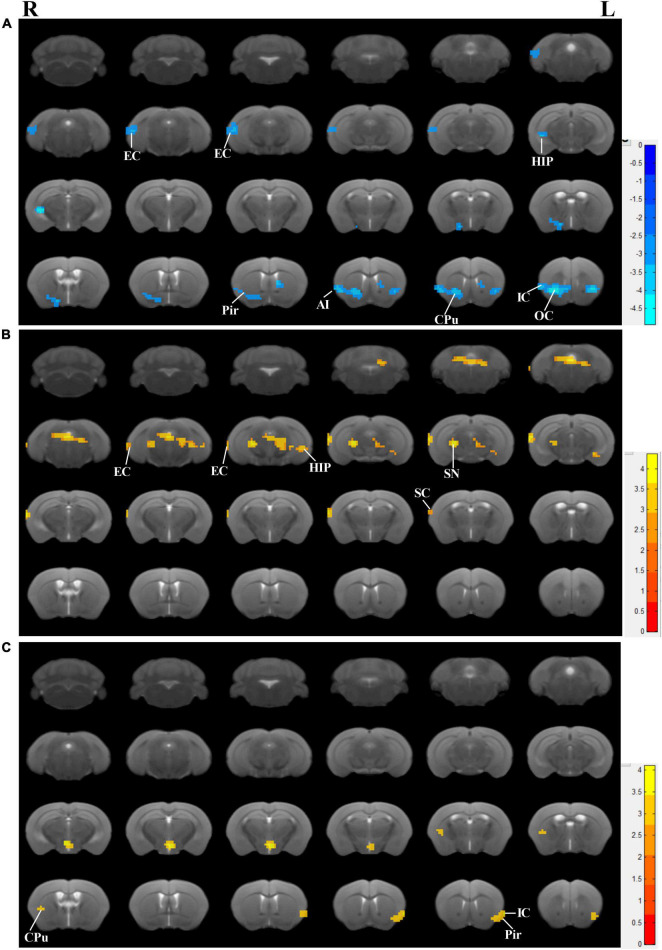
Effects of the EA-treatment on regional spontaneous activity. **(A)** Regions manifesting significant ReHo value differences between 3×Tg-AD mice and wildtype-control mice (blue, 3×Tg-AD < wildtype-control; color bar represents significance of difference), **(B)** between EA-treated and untreated 3×Tg-AD mice (red, untreated 3×Tg-AD < EA-treated; color bar represents significance of difference), and **(C)** between Non-EA-treated and untreated 3×Tg-AD mice (red, untreated 3×Tg-AD < Non-EA-treated; color bar represents significance of difference). EC, entorhinal cortex; HIP, hippocampus; Pir, piriform cortex; CPu, caudate putamen; OC, orbital cortex; IC, insular cortex; SC, somatosensory cortex; SN, substantia nigra.

Accompanied by cognitive alterations, ReHo value showed that there were brain region changes in untreated 3×Tg-AD mice compared to wildtype-control mice. A decreased regional spontaneous activity could be observed in untreated 3×Tg-AD mice in the accumbens nucleus, amygdala, anterior olfactory nucleus, auditory cortex, caudate putamen, EC, hippocampus, insular cortex, orbital cortex, piriform cortex, septal, somatosensory cortex, subiculum, temporal cortex, and visual cortex (Figure 3A).

Compared to untreated 3×Tg-AD mice, EA-treated 3×Tg-AD mice showed an increased regional spontaneous activity in the amygdala, auditory cortex, DG, dorsal raphe nucleus, EC, hippocampus, somatosensory cortex, subiculum, substantia nigra, temporal cortex, and ventral tegmental area (Figure 3B). Besides, Non-EA-treated 3×Tg-AD mice showed an increased regional spontaneous activity in the caudate putamen, insular cortex, motor cortex, orbital cortex, piriform cortex, and somatosensory cortex (Figure 3C). These results could be concluded that AD-induced chaos of brain function was attenuated by EA-treatment, which brain regions including the auditory cortex, EC, hippocampus, subiculum, and temporal cortex.

### Effects of the electroacupuncture treatment on hippocampal synaptic neurotransmission

**FIGURE 4:**
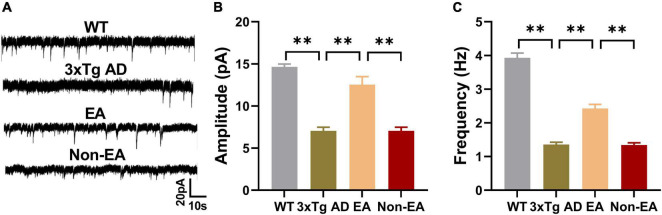
Effects of the EA-treatment on hippocampal synaptic neurotransmission. **(A)** Representative sEPSC traces of hippocampal CA1 from all mice by whole-cell voltage-clamp recording (*n* = 5 in each group). **(B)** The amplitude of sEPSC, and **(C)** the frequency of spontaneous firing of hippocampal CA1 were analyzed. Data are presented as the mean ± SEM. **P < 0.01.

The hippocampus, which is thought to be the hub of memory-related networks in AD mice, showed changes with EA-treatment. To investigate if synaptic neurotransmission of the hippocampus was influenced by the EA-treatment, we performed the whole-cell voltage-clamp recording. We compared the postsynaptic sEPSC between the 3×Tg-AD mice and wildtype-control mice by recording from hippocampus CA1, and found a significantly decreased sEPSC in the 3×Tg-AD mice, which suggested an enhanced spontaneous release of the neurotransmitter at the hippocampal terminals (Figure 4). And there was a significant difference between EA-treated and untreated 3×Tg-AD mice in the postsynaptic sEPSC of hippocampus CA1 (Figure 4), but Non-EA treatment does not affect it. The results suggested that the spontaneous release of the neurotransmitter was rescued with EA-treatment in 3×Tg-AD mice.

### Recognition memory is correlated with aberrant ReHo across the hippocampus and entorhinal cortex

**FIGURE 5:**
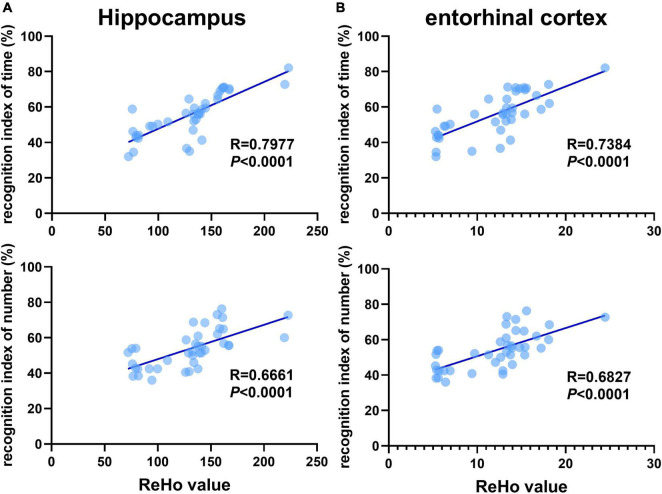
Recognition memory is correlated with aberrant ReHo across the hippocampus and entorhinal cortex. Correlational analysis between the recognition indexes (time/number) and ReHo value obtained from the **(A)** hippocampus, **(B)** entorhinal cortex (*n* = 10 in each group).

Previous studies have shown that 3×Tg-AD mice exhibit an accumulation of pathological inclusions and alterations of regional spontaneous activity in the hippocampus and EC. In addition, the hippocampus receives most of the inputs from the EC. Combining the above results, the quantitative analysis demonstrated a significantly positive correlation between differences in recognition index (time/number) and ReHo values of the hippocampus and EC, respectively (Figure 5).

### Effects of the electroacupuncture treatment on connection between the hippocampus and entorhinal cortex

**FIGURE 6:**
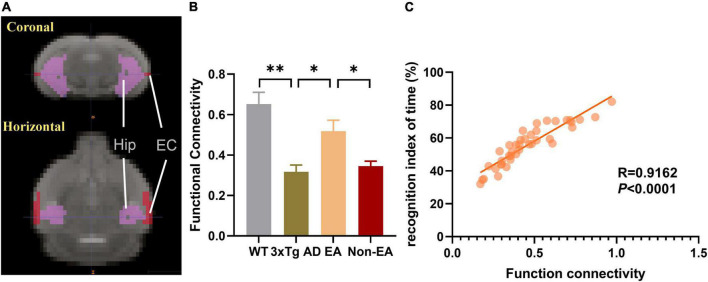
Effects of the EA-treatment on function connectivity between the hippocampus and entorhinal cortex. **(A)** Representative location image of hippocampus and entorhinal cortex. **(B)** Quantification of the function connectivity strength between the hippocampus and entorhinal cortex, **(C)** and the correlational analysis between the recognition index of time and function connectivity (*n* = 10 in each group). All data represent the mean ± SEM. *P < 0.05; ^**^P < 0.01.

Based on the above results, we took the hippocampus and EC as ROIs, FC between those was calculated within each mouse, and the mean connectivity values are presented in Figure 6. Compared to wildtype-control mice, decreased connectivity between the hippocampus and EC was observed in untreated 3×Tg-AD mice (Figure 6B). There was significantly increased connectivity with the EA-treatment. Besides, the significantly changed connection showed a difference between EA-treated and Non-EA-treated 3×Tg-AD mice. Figure 6C showed a significantly positive correlation between difference in NORT performance (recognition index of time) and FC strength between the hippocampus and EC.

**FIGURE 7:**
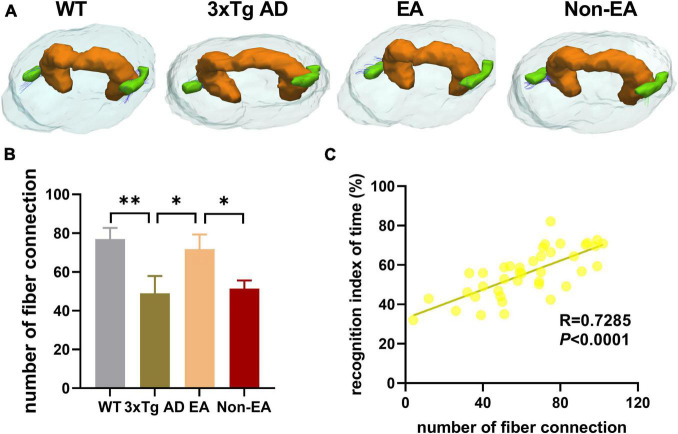
Effects of the EA-treatment on fiber connection between the hippocampus and entorhinal cortex. **(A)** Representative image of connected nerve fibers between hippocampus and entorhinal cortex in all group. **(B)** Quantification of the number of nerve fibers connection between the hippocampus and entorhinal cortex, **(C)** and the correlational analysis between the recognition index of time and the number of nerve fibers connection (*n* = 10 in each group). All data represent the mean ± SEM. *P < 0.05; ^**^P < 0.01.

Furthermore, the number of nerve fiber connections was also tested using DTI fiber tracking. As Figure 7 shown, the wildtype-control mice displayed more nerve fiber between the hippocampus and EC as compared with that of the untreated 3×Tg-AD mice. The number of nerve fiber connections between the hippocampus and EC showed an increase to ameliorate with the EA-treatment (Figure 7B). Difference between the untreated and EA-treated 3×Tg-AD mice was significant, but Non-EA-treatment presented no difference in the nerve fiber connection. Figure 7C showed a significantly positive correlation between difference in NORT performance (recognition index of time) and the number of nerve fiber connections between the hippocampus and EC.

## Discussion

The current study explored the effects of EA-treatment on recognition memory and neural activity connections in 3×Tg-AD mice. The behavioral test showed that EA-treatment ameliorated recognition memory impairment in 3×Tg-AD mice. The regional homogeneity was measured by rs-fMRI and is known to reflect alterations in local neuronal integration. Our study showed that EA-treatment increased the ReHo value of local neuronal integration in the Hip, EC, and other brain regions. Besides, the local neuronal integration is thought to be caused by synaptic dysfunction in early AD. Further, we observed increased synaptic neurotransmission in hippocampal CA1 with EA-treatment. The EC-Hip is a critical circuit for object recognition memory. Moreover, we demonstrated that EA-induced recognition memory behavior was associated with aberrant local neuronal integration in the Hip and EC. Further analysis revealed that EA-treatment increased FC and the number of nerve fiber connections between the EC and Hip, suggesting hippocampus-cortex network modulation induced by EA.

3×Tg-AD mice express mutant alleles of human APP, PS1, and Tau genes that correspond to the coexistence of most APP and Tau genes in human AD currently found in clinical practice, which simulates the pathological process (Oddo et al., 2003). The mice investigated here in the early AD states, developed memory deficit, together with aberrant local neuronal integration and long-range connectivity (Liu et al., 2018; Manno et al., 2019). In the current study, the results we assessed showed a decrease in locomotor and recognition memory in 3×Tg-AD mice. It can be considered that AD model mice have a deficit in memory encoding and/or storage at this stage, which is consistent with the clinical manifestation of AD patients. Compared with the healthy elderly, in previous studies, it was difficult to establish elaborative encoding of target information or context in the early AD populations (El Haj et al., 2015a,b).

Electroacupuncture treatment is recognized as an effective therapeutic of TCM to alleviate cognitive disorders. Previous animal experiments have suggested that EA-induced neuromodulation in AD model mice included regulation of Aβ protein, tau phosphorylation, neuroinflammation, brain plasticity, neuron apoptosis, mitochondrial activity, and blood–brain barrier function. However, the role of EA-treatment in preventing cognitive impairment in the early AD stage remains unclear. In our studies, we found that EA-treatment was able to improve the object recognition memory in 3×Tg-AD mice, which is in accordance with previous finds (Cai et al., 2019). Meanwhile, there is no alteration in locomotor activity with EA-treatment, which may be attributed to the specific role of acupoints inserted. According to the TCM theory, EA at DU20 and DU24 could enhance brain function and improve cognitive function. Previous clinical trials showed that the combination of DU20 with other acupoints downregulated the level of Aβ and cognitive function in AD patients (Yu et al., 2019). Besides, the role of the specific acupoints could also explain why our results showed no behavioral difference between the EA and Non-EA on AD mice, consistent with our previous studies (Lin et al., 2018; Li et al., 2020).

The synchronized neuronal activities and their network connectivity are essential for memory information transmission and processing, which are affected in AD populations (Uhlhaas and Singer, 2006; Shah et al., 2016). Besides, the Hip is consistently affected by neuronal degeneration, which is the hub of networks responsible for the early memory impairment in AD (Najm et al., 2019; Dong et al., 2021). The effect of EA-treatment on brain regions such as the hippocampus in AD patients has been confirmed to some extent. Clinical trials found significant neuronal activity changes in the right middle cingulate cortex, right inferior frontal gyrus, right hippocampus, and right inferior temporal gyrus of AD patients by EA (Zheng et al., 2018). Besides, our previous study found that EA at DU20 acupoint enhances glucose metabolism in the cortex, hippocampus, and other brain regions of AD mice (Liu et al., 2017). In the present study, our ReHo analysis revealed that EA-treatment increased synchronous activity in the Hip and brain regions (EC, subiculum, temporal cortex) known to be affected in AD patients, which is consistent with previous studies. In addition, brain regions including the subiculum, temporal cortex, and orbitofrontal cortex are closely associated with attention and decision-making, and the subiculum is regulated by the EC-Hip circuit. Moreover, researchers have confirmed the delay-dependent contribution of the CA1 region to object memory (Ásgeirsdóttir et al., 2020; Cinalli, et al., 2020). Our results revealed that spontaneous release of the neurotransmitter was rescued with EA-treatment in 3×Tg-AD mice. To summarize, we can speculate that the hippocampus is a key brain region for EA-treatment to improve memory function in AD model mice.

Furthermore, EC and Hip dysfunction are thought to be possible causes of initial memory symptoms (Van Hoesen et al., 1991). They are the earliest regions affected by AD, and the EC conveys spatial/contextual and object information to the hippocampus (van Strien et al., 2009; Knierim et al., 2014). Previous observations have revealed that coherent oscillatory activity in EC-Hip was correlated with object recognition memory performance (Salimi et al., 2022). Consistent with previous studies, the result in the present study has shown that EC and Hip are correlated with recognition memory performance. The clinical trials found that the connecting fibers between the hippocampus and related brain regions of AD patients were damaged and their functional connection strength decreased, which was closely related to the cognitive decline of AD (Quan et al., 2020; Dautricourt et al., 2021). Moreover, deep brain stimulation at EC affected increased resting-state FC and an obvious information flow from the EC to the Hip (Jiang et al., 2022). We found that the EA-treatment could increase the connecting fibers and functional connection strength between EC and Hip, indicating that the effects of EA-treatment might rely on EC–Hip network modulation mechanisms.

Importantly, there are important limitations and further investigation for the present study. Firstly, given the high incidence and earlier-onset features of AD cases in women, however, only male 3×Tg AD mice were used in our experiments, which limits our interpretation for both sexes. In addition, it is necessary to combine multiple high-resolution methods to detect the roles of different subregions of the hippocampus and EC, and the network connectivity between the hippocampus and other brain regions also needs to be further studied. Moreover, further experiments are necessary to determine the role of the EC -hippocampal circuit in recognition memory deficit in 3×Tg-AD mice with EA-treatment and to investigate the biomolecular mechanism(s) at work.

Conclusively, the data in the present work recapitulated that EA alleviated object recognition memory deficit associated with ameliorated neuronal activities and network connection in the 3×Tg-AD mice model. Our results suggested that the effect of EA- treatment may be dependent on the EC-Hip connection. EA may be a potentially translatable strategy for recognition memory impairment in AD patients.

## Data availability statement

The raw data supporting the conclusions of this article will be made available by the authors, without undue reservation.

## Ethics statement

The animal study was reviewed and approved by the Fujian University of Traditional Chinese Medicine Animal Experiment Ethics Committee and Authority.

## Author contributions

JH and BL designed the study. JH, BL, and LZ wrote the manuscript. BL, XC, and LZ performed the experiments. XY, ZL, CR, and TW analyzed the data. All authors have read and approved the final manuscript.

## Footnotes

1
http://www.fil.ion.ucl.ac.uk/spm

2
http://www.restfmri.net/forum/DPARSF

## Abbreviations

AD: Alzheimer’s disease
EA: electroacupuncture
Hip: hippocampus
MCI: mild cognitive impairment
DTI: diffusion tensor imaging
EC: entorhinal cortex
OFT: open field test
NORT: novel object recognition test
sEPSC: spontaneous postsynaptic currents
PCR: polymerase chain reaction
rs-fMRI: resting-state functional magnetic resonance imaging
T2WI: T2-weighted images
EPI: echo-planar imaging
FC: function connectivity
ROIs: regions of interest
ACSF: artificial cerebrospinal fluid
KCC: Kendall’s coefficient of concordance.
