# Supplementary figures and images for: Modulation of entorhinal cortex–hippocampus connectivity and recognition memory following electroacupuncture on 3×Tg-AD model: Evidence from multimodal MRI and electrophysiological recordings
Source: Front Neurosci. 2022 Jul 29;16:968767. doi: 10.3389/fnins.2022.968767 (PMC9372370; doi:10.3389/fnins.2022.968767)

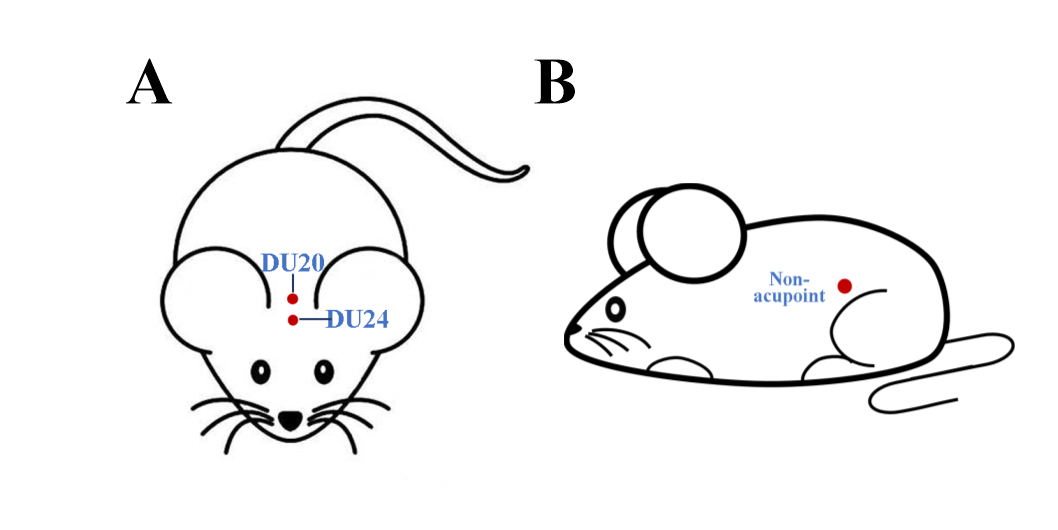

Supplement: Supplementary file 1 [file Image_1.TIF]
